# Impact of essential oil and probiotics supplementation on growth performance, serum biomarkers, antioxidants status, bioenergetics and histomorphometry of intestine of Nile tilapia fingerlings challenged with *Aeromonas veronii*

**DOI:** 10.1186/s12917-024-04433-w

**Published:** 2025-01-07

**Authors:** Walaa S. Raslan, Ahmed Shehab, Aya F. Matter, Hadeer A. Youssuf, Omar Ahmed Farid, Ahmed Sabek, Yasmeen Magdy, Amgad Kadah

**Affiliations:** 1 Department of Physiology, Faculty of Veterinary Medicine, Benha University, Moshtohor, Toukh, Qalyubia 13736 Egypt; 2 Department of Nutrition and Clinical Nutrition, Faculty of Veterinary Medicine, Benha University, Moshtohor, Toukh, Qalyubia 13736 Egypt; 3 Department of Aquatic Animal Medicine, Faculty of Veterinary Medicine, Benha University, Moshtohor, Toukh, Qalyubia 13736 Egypt; 4 Department of Physiology, National Organization for Drug Control and Research, Giza, Egypt; 5 Department of Hygiene and Veterinary Management, Faculty of Veterinary Medicine, Benha University, Moshtohor, Toukh, Qalyubia 13736 Egypt; 6 Department of Anatomy and Embryology, Faculty of Veterinary Medicine, Benha University, Moshtohor, Toukh, Qalyubia 13736 Egypt

**Keywords:** Feed supplementations, (*Oreochromis niloticus*), Growth performance, Oxidative stability, *Aeromonas veronii*

## Abstract

**Background:**

Probiotics and essential oils feed supplements are widely used in the aquaculture sector. This study was conducted to evaluate the effects of dietary supplementation with probiotics, essential oils and their combination on growth performance, serum biochemical parameters, antioxidant capacity, resistance against *Aeromonas veronii*, and intestinal histomorphology of Nile tilapia (*Oreochromis niloticus*). A total of 360 *O. niloticus* fingerlings were randomly assigned to four groups (3 replicates/ group; each replicate contains 30 fish) based on the different dietary treatments. The first group was fed a basal control diet (G1), the second group was fed a basal diet supplemented with 0.015% probiotic (Klu-zetar^®^) (G2), the third group was fed a basal diet with 0.015% essential oil (ACTIVO^®^) (G3), and the fourth group was fed a basal diet mixed with 0.015% Klu-zetar^®^ and 0.015% ACTIVO^®^, (G4) for 6 weeks. At the end of the trial fish were intraperitoneally injected with pathogenic bacteria *Aeromonas veronii* and the fish mortality rate was recorded for 7 days post infection.

**Results:**

The results revealed that using probiotics and or essential oils in Nile tilapia diets improved growth performance, reduced oxidative stress, enhanced immunity, maintained intestinal integrity, and enhanced resistance to pathogenic infection (*P* ≤ 0.05).

**Conclusions:**

It is concluded that the use of probiotics and/ or essential oils enhance the overall outcomes of Nile tilapia, so it is highly recommended to be used in aquaculture management.

## Background

*Oreochromis niloticus*, also known as Nile tilapia, is a popular freshwater aquaculture fish species raised in numerous countries around the world [1–3]. Nonetheless, by 2030 it is anticipated to account for around 62% of all aquaculture production worldwide [4]. Bacterial infections in tilapia aquaculture can cause a large amount of mortality. *Aeromonas spp*. are opportunistic and motile microbes that frequently infect fish with impaired immune systems because of adverse environmental factors, inadequate nutrition, high stocking densities, excessive handling, prolonged transportation, and mechanical injury [5–7].

Fish farmers are very concerned about controlling fish infections, which is commonly achieved by using antibiotics, which the EU has outlawed [8]. From an environmental and public health standpoint, the use of conventional antibiotics as growth promoters is considered inappropriate since the European Union imposed penalties on the practice in the mid-2000s [9]. The fish production chain suffers financial losses as a result of these sanctions, which also directly affect the amount and quality of animal protein produced and the risk of mortality [10].

In recent years, the importance of disease prevention has grown, particularly with regard to the substitution of feed additives for chemical additives and veterinary drugs. Probiotics and phytogenic compounds are two common feed additions [11–15].

For instance, it has been reported that *Bacillus subtilis* is non-pathogenic and non-toxic [16] and can improve aquaculture in a number of ways, including by increasing fish productivity [17–19], secreting antimicrobial agents that kill pathogens [20, 21], inhibiting the expression of virulence genes, lytic enzyme synthesis, releasing bacteriocin, and forming organic acids [22]. Moreover, probiotics have been deemed generally recognized as safe (GRAS) for consumption by humans and animals by the Food and Drug Administration [21]. Because essential oils (EOs) have a positive effect on growth promotion, health, and disease resistance in fish, they have drawn more attention as feed additives in aquaculture [23].

Antibacterial, antioxidant, and growth-promoting properties may be found in EOs derived from plants [7, 23, 24]. Because of the synergistic impact of its ingredients, EO mixes have greater active capacity and can be active against a wide spectrum of species, which is why they are used. The advantages of employing these mixes in a variety of fish species are further supported by the vast array of phototherapeutics available and the possible quantity of distinct bioactive molecules [25].

EOs have been considered safe by the Food and Drug Administration (FAD) since 2004. To the best of our knowledge, the effectiveness of dietary supplementation with Klu-zetar^®^ probiotic and ACTIVO^®^ essential oil on growth performance, serum biomarkers, antioxidant status, and against *Aeromonas veronii* infection in juvenile Nile tilapia has not been studied. Hence, this study was conducted to assess the impact of commercial probiotic and/or essential oil products on growth performance, antioxidants, histomorphometry of the small intestine, and response of *O. niloticus* to *A. veronii* infection challenge.

## Materials and methods

### Ethics statement

The current feeding trial was carried out in the aquaculture Research unit, Department of Physiology, Faculty of Veterinary Medicine Benha University, Egypt. All experimental protocols, management conditions, handling, and sampling were approved by the Institutional Animal Care and Use Committee Research Ethics Board, Faculty of Veterinary Medicine, Benha University, under the ethical number (BUFVTM 40-09-23).

### Fish and diet

Three hundred and sixty *O. niloticus* fingerlings weighing an average of twenty-five to sixty-five grams on average were purchased from a licensed private fish farm in the Kafr El-Sheikh Governorate, Egypt. The fingerlings were brought to the laboratory in double polyethylene bags that supplied oxygen. Upon arrival, the fish were checked for abnormal movement, faded or darkened pigmentation, skin lesions, fin and tail erosion, and external fungi or parasites. Before being given a commercial tilapia diet (30% CP), fish were kept in 500-liter aerated fiberglass tanks for two weeks to allow them to get acclimated to aquarium settings. The water parameters were adjusted in the order given by [26] (water temperature: 28 °C; oxygen concentration: 6 mg/L; ammonia concentration: 0.53 mg/L; pH: 7).

The diets were created to meet the dietary requirements of Nile tilapia, according to the National Research Council. The feeding study’s nutritional composition is displayed in Table 1. The ingredients used for the fish food were thoroughly mixed and left for 15 min. Then, water and oil were added to create a moist, doughy mass. Next, without using any steam, the dough mass was pelleted, producing 2 mm-diameter sinking pellets. Using the procedures outlined in [27], the pellets were lastly dried at ambient temperature and kept in sterile, clean plastic bags at -20 °C until needed. The additives used in this study were Klu-Zetar^®^ probiotic and ACTIVO^®^ essential oil. The probiotic product contains *Bacillus subtilis* (ATCC-PTA 6367) 1.3 × 10^11^ CFU/kg, *Bacillus subtilis* (DSM 5750) 0.13 × 10^11^ CFU/kg, *Bacillus licheniformis* (DSM 5749) 1.3 × 10^11^ CFU/kg, and *Clostridium butyricum* (FREM BP-2789) 1 × 10^8^ CFU/kg. ACTIVO (Grasp industriae Comercio Ltda, Brazil, imported by EW Nutrition GmbH, Germany), essential oil product contains carvacrol 50 g/kg, thymol 2 g /kg, cineol 10 g/kg, cinnamaldehyde 0.5 g/kg, and capsaicin 10 g/kg.

**Table 1:** Ingredients and composition of the experimental basal diet

Ingredients	%
Yellow corn	15.9
Soybean meal (44% protein)	29.5
Corn gluten (60% protein)	6.
Fish meal	12
Rice bran	11
Wheat bran	10
Wheat flour	4
Soybean oil	2
Molasses	2
Choline chloride	0.075
Common salt	0.15
Vitamin and mineral premix**	0.35
Vitamin C	0.025
**Nutrient specification**	%
Crude protein	30.
Crude lipids	5.57
Crude fiber	5.12
Calcium	1
Total phosphorus	0.62
Lysine Methionine Threonine Cystine + Methionine Arginine	1.63 0.60 1.12 1.04 1.81
Gross energy, kcal kg^− 1^ diet	4050

** Premix provided each 1 kg of feed with Biotin = 0.025 mg; Folic Acid = 1 mg; Niacin = 20 mg; Pantothenic acid = 8 mg; Vitamin A = 7000 IU; Vitamin B1 = 1 mg; Vitamin B12 = 0.01 mg; Vitamin B2 = 4 mg; Vitamin B6 = 1 mg; Vitamin D = 1400 IU; Vitamin E = 10 mg; Vitamin K3 = 3 mg; Cobalt = 0.01 mg; Copper = 10 mg; Iodine = 0.05 mg; Iron = 15 mg; Manganese = 40 mg; Selenium = 0.01 mg; Zinc = 40 mg. The 1st group fed the basal control diet, and the other groups (2nd, 3rd, and 4th ) were fed basal diet supplemented with 0.015% probiotic (Klu-zetar^®^), 0.015% essential oil (ACTIVO^®^) and mixture of 0.015% Klu-zetar^®^ and 0.015% ACTIVO^®^, respectively

### Experimental design

In four nutritional treatment groups, a total of 360 O. niloticus fingerlings were randomly assigned to 500 L tanks (triplicate design, 90 fingerlings per group, 30 fingerlings each replication).The first group was given the basal control diet (G1), while the subsequent groups received a basal diet plus 0.015% probiotic (Klu-zetar^®^) (G2), a basal diet plus 0.015% essential oil (ACTIVO^®^) (G3), and (G4) fed on a basal diet supplemented with both 0.015% Klu-zetar^®^ and 0.015% ACTIVO^®^. Twice a day, at 8:00 a.m. and 4:00 p.m. for 6 weeks, fish were hand fed at a rate of 5% of their body weight. Water was partially exchanged 3 times weekly.

### Growth parameters

Every two weeks, the fish’s body weight was measured. Before being weighed, the fish in each tank were dried with sterile, clean filter paper to eliminate any extra water, and they were fasted for six hours. The amount of leftover food was collected, allowed to air dry, and the quantity collected was subtracted from the amount provided to determine the daily feed consumption. A previously published approach [26] was used to quantify growth performance, which included initial body weight, final body weight (FBW), and weight gain (WG).

Feed conversion rate (FCR)=feed intake (g)/weight gain (g), as previously described [28].

Weight gain rate (%) = (Average final body weight− Average initial body weight) \Average of initial body weight.

Average daily gain (ADG) = final body weight- initial body weight/ time of the trial.

Specific growth rate (SGR) = (Ln Final weight − Ln Initial weight)/ (No of days in trial) × 100.

Condition factor (CF) = (W/L^3^) x 100.

### Sampling

The 6-week feeding experiment was followed by the random selection of three fish per replicate (nine fish/group) and their excessive use of an anesthetic solution (MS 222; acquired from Sigma Aldrich Chemicals, CO, USA) diluted 1:4000 in dechlorinated water for two minutes. Using 3 mL size syringes, the caudal vein and heart were punctured in order to exsanguinate Nile tilapia. To separate the serum, the blood was left to coagulate for two hours at 4 °C as recommended by [29]. The serum was centrifuged at 3500 x g for 25 min at 4 °C after separation. Prior to a more thorough examination, the samples were kept in storage at -80 °C. Liver samples were taken after the fish was dissected and put in microcentrifuge tubes with phosphate-buffered saline (PBS) in them. After that, liver samples were homogenized using an electrical homogenizer (Heidolph, Germany) at a ratio of 1:10 (w/v) in chilled PBS with a pH of 7.4. The homogenates undergo a 15-minute, 4000 x g centrifugation at 4 °C while being kept on ice. The supernatants were kept at -20 °C [30].

### Serum biochemical analysis

Using commercial kits from the Diamond Diagnostics Company, Egypt, cortisol concentrations, AST, and ALT levels were determined spectrophotometrically at 340 nm, as reported in [31, 32]. Spectrophotometric analysis was used to determine the blood total cholesterol level in accordance with [33]. Serum triglycerides was determined spectrophotometrically according to [34]. The determination of serum high- and low-density lipoprotein concentrations were done as described by [35].

### Estimation of oxidative stress biomarkers and antioxidants in liver

Using an HPLC (Agilent HP 1200 Series Apparatus, USA) system, the liver’s concentrations of malondialdehyde (MDA), reduced glutathione (GSH), oxidized glutathione (GSSG), and 8-hydroxy-deoxyguanosine (8-OHdG) were determined. The methods for determining MDA that were previously stated were used [36, 37]. The same HPLC method was used to assess the thiol compositions of reduced and oxidized glutathione in liver tissues. In contrast to this case, the HPLC was equipped with a Bondapak column (30 cm 3.9 mm C18l) and was loaded with a mobile phase comprising pH 3.5, 0.005 M tetrabutylammonium phosphate, 13% methanol, and 0.0025 M sodium phosphate buffer. Hepatic Co Q10 analysis was made possible by extracting liver sample by centrifuging 1-propanol at 2000 x g for 10 min at 4 C.

The coQ10 measurement was carried out in accordance with [38]. A series of C18 reversed-phase columns (Supelco, 5 p.m., I.D. 0.46 25 cm) were used to separate the amounts of 8-hydroxy-20-deoxyguanosine (8-OHdG) in the hepatic tissues at a wavelength of 245 nm and a flow rate of 0.68 mL/min. Spectrophotometry was used to measure the activity of superoxide dismutase (SOD). In a nutshell, five milliliters of cold PBS (1:5 dilution) were mixed with one gram of tissue. Each of these samples underwent a 15-minute centrifugation at 1,968 x g and 4 °C. Up until biochemical assays on superoxide dismutase (SOD) activity were conducted, supernatants were collected and kept at 20 °C. The method of testing involves measuring how well the SOD enzyme inhibits pyrogallol autoxidation for a period of two minutes at a time. Nitric Oxide (NO) (mol/g) was detected in accordance with [39].

### Determination of ATP, ADP and AMP contents in liver

The liver’s ATP, ADP, and AMP contents (g/g tissue) were measured using HPLC. An Ultrasphere ODS EC 250 × 4.6 mm column was used for mobile phase separation. The detecting wavelength was 254 nm, and the flow rate was 1.2 mL/min. Phase B consisted of 100% acetonitrile, whereas phase A consisted of 0.06 mol/L and 0.04 M K2HPO4 dissolved in deionized water and calibrated to pH 7.0 with 0.1 M KOH. Each specimen’s ATP, ADP, and AMP chromatograms were identified through matching them to standards supplied by Sigma Aldrich [40].

### Histological examination

#### Light microscopy (LM)

Fish gut fragments were removed and immediately preserved in 10% buffered neutral formalin. They were then dried in alcohol, washed in xylene, embedded in paraffin, and sliced into thin, 5 μm thick slices. The sections underwent hematoxylin and eosin (H&E) staining. Procedures and techniques for fixation and staining have been detailed by [41]. The stained slices were examined on a Leica DM 3000 LED computerized light microscope.

#### Scanning electron microscopy (SEM)

After thoroughly cleaning the intestinal mucosal surface with normal saline to get rid of any food particles, tiny fragments of fresh specimens were cut and preserved in glutaraldehyde (pH 7.4) for three hours at 4 °C. The samples underwent three PBS washes (10 min each), a post-fixation in 1% osmium tetraoxide for 30 min at room temperature, a series of ethyl alcohol dehydration (30, 50, 70, 90%, and 100% alcohol), and an acetone infiltration. Using a Jeol-JSM-5300 LV scanning electron microscope (Tokyo, Japan) set to 20 KV at the electron microscopy facility at Alexandria University in Egypt, the intestinal villi in coded samples were seen. For thirty minutes, the tissues were submerged in each solution. Samples were placed on aluminum rods and gold-coated to a thickness of 0.04 lm in a sprayer-coating equipment (JFC-1100 E) after being dried in a Samdri-PVT-3B^®^ (Tousmisis, Rockville, USA), a critical point drier, using liquid carbon dioxide. A Jeol-JSM-5300 LV scanning electron microscope (Tokyo, Japan) operating at 20 KV was used to observe the intestinal villi in the encoded samples at Alexandria University in Egypt.

#### Bacteria and challenge experiment

Following a six-week feeding trial, *Aeromonas veronii* at a concentration of 1.0 × 10^8^ CFU/ml was administered intraperitoneally (I/P) to ten fish per replication (30 fish/group) using a 0.1^-ml^ injection, as per the protocol reported in [42]. Daily observations of the mortality rate were made for seven days. The formula for calculating the mortality percentage was as follows: total mortality / total number of infected fish × 100. Relative percentage of survival (RPS) = 100 [1 − (treatment-specific mortality / control-specific mortality)]. Koch’s postulates test was used to confirm that the fish were infected with the bacteria used by making re isolation and complete re identification to the bacteria used.

#### Statistical analysis

Data analysis was done using SPSS version 22. Analysis of variance (ANOVA) was used to assess the data. A Shapiro-Wilk test was used to determine if the distribution of the data was normal. The data were presented using means and standard errors. A difference in the data was defined as *P* ≤ 0.05.

## Results

### Growth performance

The effects of probiotics and/or essential oils are displayed in Table 2. The growth parameters (FW, WG, SGR%, FCR, ADG, body length and CF) were significantly (*P* < 0.05) improved in all dietary treatment groups in comparison with the control group. In addition, the best growth performance parameters were recorded in G2, in which fish were fed on a diet supplemented with probiotics only.

**Table 2:** Growth performance of Nile tilapia fed diets supplemented with probiotic and/or essential oil

Growth performance parameters	G1	G2	G3	G4	SEM	*P*- value
Initial body weight(g)	25.02	25.07	24.97	24.95	0.057	0.894
Final body weight(g)	37.61^c^	72.06^a^	63.25^a^	53.67^b^	2.54	0.001
Weight gain(g)	12.59^c^	46.99^a^	38.28^a^	28.27^b^	2.54	0.001
Final length (cm)	12.86^c^	14.71^a^	14.13^a^	13.39^c^	0.17	0.001
SGR(%/day)	1.52^c^	1.8^a^	1.74^a^	1.67^b^	0.02	0.001
Weight gain rate	50.30^c^	187.23^a^	153.18^b^	115.15^b^	10.05	0.001
FCR	2.17^a^	1.27^b^	1.43^b^	1.56^b^	0.09	0.001
ADG (g/day)	0.42^c^	1.56^a^	1.27^b^	0.95^bc^	0.084	0.001
CF	1.88^b^	2.02^a^	2.00^a^	1.93^ab^	0.018	0.027

Means with different superscripts letters in the same raw are significantly different at *p* < 0.05. G1, a basal diet; G2 = a basal diet supplemented with 0.015% probiotic (Klu-zetar^®^); G3, a basal diet supplemented with 0.015% essential oil (ACTIVO^®^); G4, a basal diet supplemented with mixture of 0.015% Klu-zetar^®^ and 0.015% ACTIVO^®^, respectively. SGR, Specific growth rate; FCR, Feed conversion ratio; ADG, Average daily gain; CF, condition factor

### Biochemical parameters

The current results revealed that the different dietary supplementations had no significant effect on AST, ALT, TC, TG, and LDL values. The lowest value of HDL was recorded in the 4th group fed a diet containing a combination of probiotics and essential oils. Additionally, serum cortisol level was markedly decreased (*p* < 0.05) in G3 and G4 relative to those in G2 and G1, respectively (Table 3).

**Table 3:** Effect of different dietary treatments on serum biochemical indices of Nile tilapia

Serum biochemical indices	G1	G2	G3	G4	SEM	*P*- value
AST (U/L)	37.69	39.78	40.49	33.97	1.12	0.15
ALT (U/L)	55.06	44.75	52.68	49.69	1.79	0.2
TC (mg/dl)	131.68	127.39	134.42	106.6	5.33	0.23
TG (mg/dl)	117.98	120.20	113.42	101.58	5.028	0.62
LDL (mg/dl)	60.00	56.18	52.18	49.04	4.15	0.857
HDL (mg/dl)	47.66^ab^	46.66^ab^	58.33^a^	36.00^b^	2.83	0.015
Cortisol (µg/dl)	37.61^a^	35.30^ab^	33.74^b^	27.33^b^	1.45	0.03

Means with different superscripts letters in the same raw are significantly different at *p* ≤ 0.05. G1, a basal diet; G2 = a basal diet supplemented with 0.015% probiotic (Klu-zetar^®^); G3, a basal diet supplemented with 0.015% essential oil (ACTIVO^®^); G4, a basal diet supplemented with mixture of 0.015% Klu-zetar^®^ and 0.015% ACTIVO^®^, respectively. ALT: Alanine aminotransferase; AST: Aspartate aminotransferase; TC: Total Cholesterol; TG: Triglycerides; HDL, high density lipoprotein; LDL, low density lipoprotein

### Oxidative stress biomarkers

Table 4 demonstrates a clear decrease (*p* < 0.05) in MDA, GSSG, and NO levels across all dietary treatment groups as compared with the control group. Fish that were fed diets that included a combination of essential oil, and probiotics demonstrated the lowest concentrations (*p* < 0.05) of nitric oxide (NO), oxidized glutathione (GSSG), malondialdehyde (MDA), and 8-hydroxy-2-deoxyguanosine (8-OHdG) in their livers. SOD, GSH, and CoQ10 values were significantly higher (*p* < 0.05) in G2, G3, and G4. ATP and AMP levels in the liver were noticeably higher within G3 and G4. Moreover, when ADP concentrations in each group were compared to the control, no discernible changes were found.

**Table 4:** Effect of probiotic and essential oil on antioxidant status of Nile tilapia

Items	G1	G2	G3	G4	SEM	*P*- value
MDA (nM/g)	55.17^a^	49.86^b^	45.60^b^	35.01^c^	2.31	0.001
SOD (nM/min/g)	43.30^b^	52.89^a^	54.99^a^	55.36^a^	1.81	0.01
GSH(nM/g)	2.82^b^	3.63^a^	3.87^a^	4.82^a^	0.18	0.013
GSSG(nM/g)	0.33^a^	0.30^ab^	0.28^b^	0.27^b^	0.008	0.04
8-OHdG (nM/g)	161.33^a^	151.37^a^	137.93^ab^	118.10^b^	5.97	0.027
CoQ_10_ (nM/g)	5.12^c^	7.05^b^	7.61^ab^	8.19^a^	0.36	0.001
NO(nM/g)	0.281^a^	0.246^b^	0.216^b^	0.207^c^	0.0096	0.003
ATP (µg/g)	55.20^b^	57.28^b^	66.22^a^	67.54^a^	1.97	0.02
ADP (µg/g)	13.96	13.46	14.79	15.75	0.43	0.27
AMP (µg/g)	27.11^b^	27.56^b^	30.06^a^	32.01^a^	0.66	0.03

Means with different superscripts letters in the same raw are significantly different at *p* ≤ 0.05. G1, a basal diet; G2 = a basal diet supplemented with 0.015% probiotic (Klu-zetar^®^); G3, a basal diet supplemented with 0.015% essential oil (ACTIVO^®^); G4, a basal diet supplemented with mixture of 0.015% Klu-zetar^®^ and 0.015% ACTIVO^®^, respectively. MDA, malondialdehyde; SOD: superoxide dismutase; nM: nanomole; GSH: reduced glutathione; GSSG: oxidized glutathione; CoQ_10_: Coenzyme Q10; 8-OHdG: 8-hydroxy-2-deoxyguanosine; ATP: adenosine triphosphate; ADP: adenosine diphosphate; AMP: adenosine monophosphate; NO: nitric oxide

### Histological findings

**Fig. 1:**
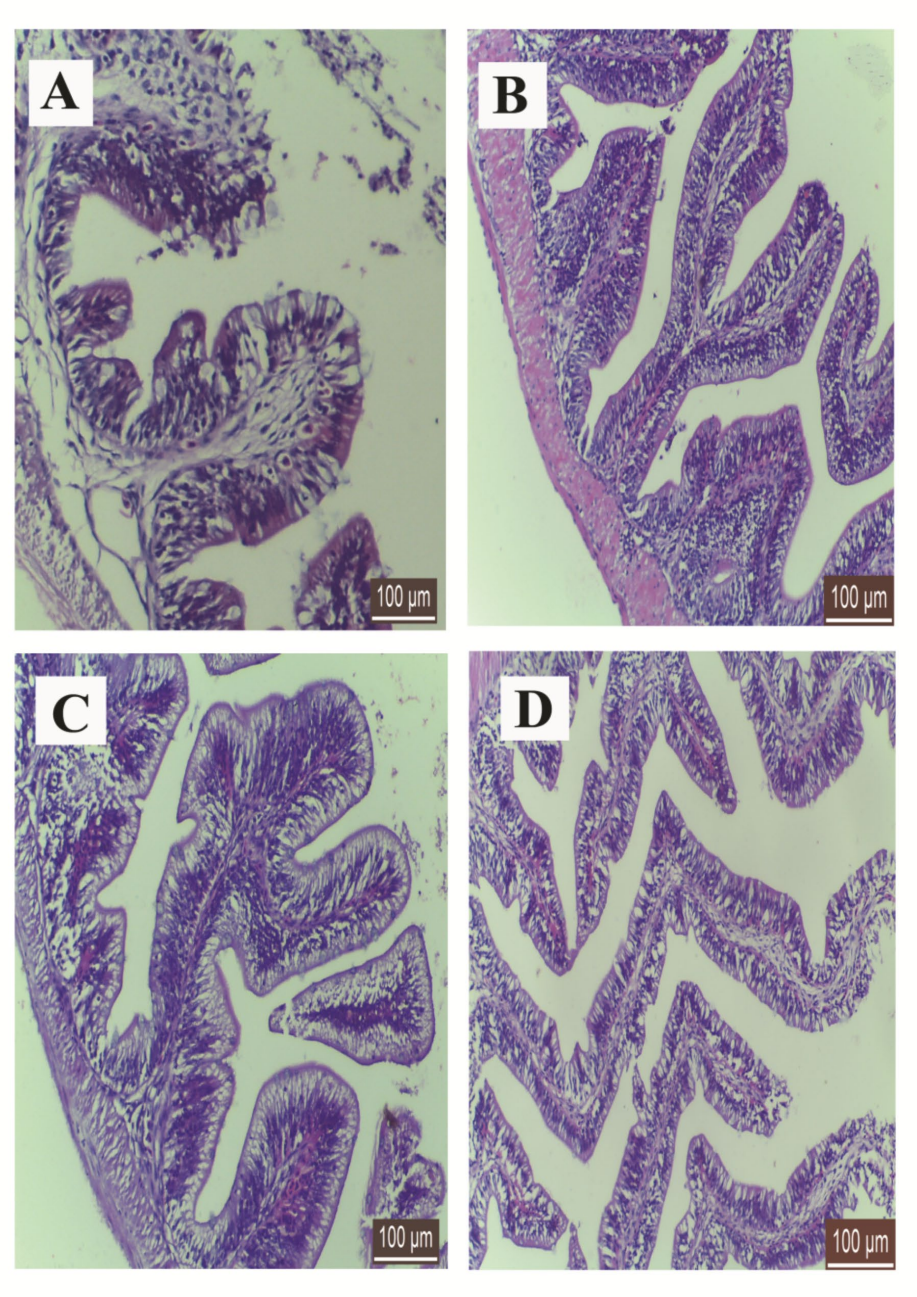
Histological sections of *O. niloticus* mid intestines showing gradual increase of length of the intestinal villi in different groups of study. H&E stain, **A**: control group, **B**: probiotic group, **C**: essential oil group and **D**: mix group. Bar indicates magnification

**Fig. 2:**
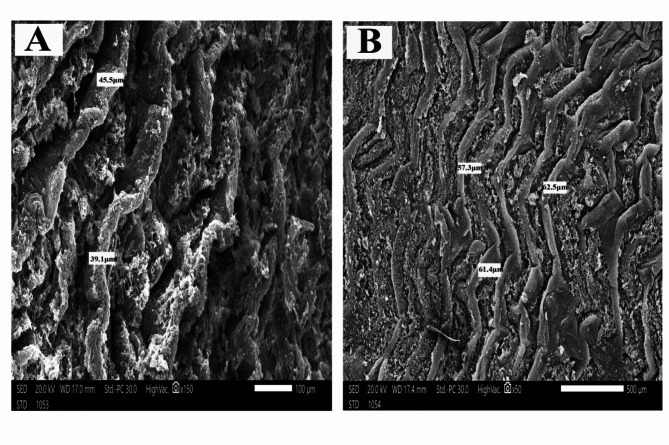
Scanning electron micrograph *O. niloticus* mid intestine of that fed the control diet. **A**: high magnification and **B**: low magnification, bar indicates magnification

**Fig. 3:**
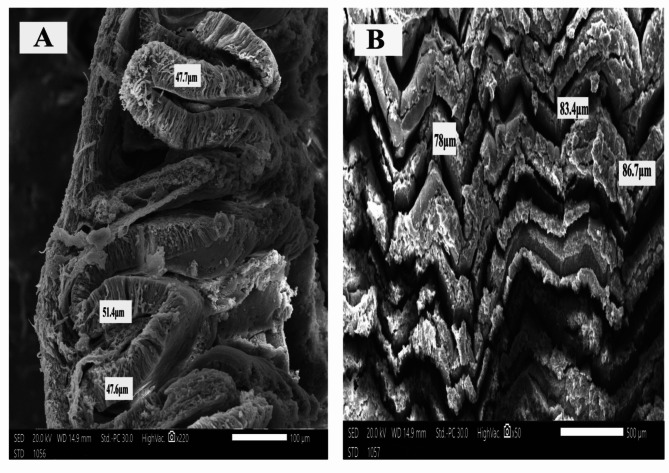
Scanning electron micrograph of *O. niloticus* mid intestine of that fed Klu-zetar. **A**: high magnification and **B**: low magnification, bar indicates magnification

**Fig. 4:**
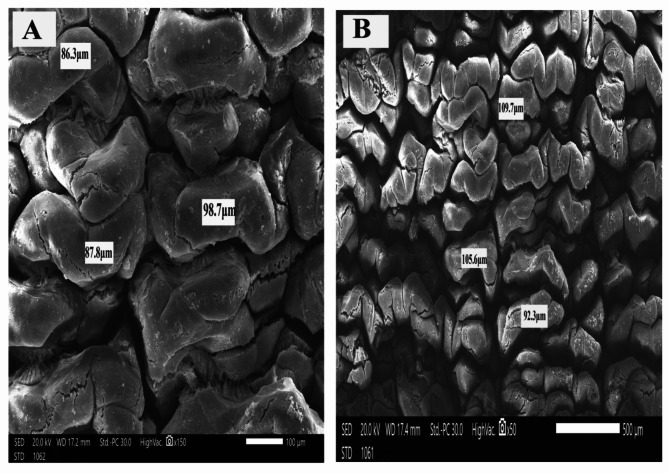
Scanning electron micrograph of *O. niloticus* mid intestine of that fed ACTIVO. **A**: high magnification and **B**: low magnification, bar indicates magnification

**Fig. 5:**
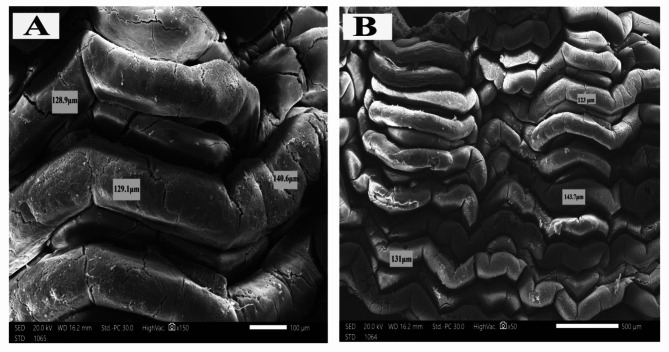
Scanning electron micrograph of *O. niloticus* mid intestine of that fed mixture of Klu-zetar and ACTIVO. **A**: high magnification and **B**: low magnification. Bar indicates magnification

The histological sections of *O. niloticus* mid intestine of all groups under a light microscope are shown in (Fig. 1). The scanning electron microscopy of *O. niloticus* mid intestine of different dietary groups is shown in Figs. 2, 3, 4 and 5. The width and length of the mid intestine of different groups are displayed in Table 5.

**Table 5:** Effect of probiotic and essential oil on average width and length of the intestinal villi (mid intestine) of Nile tilapia

Intestinal villi	G1	G2	G3	G4	SEM	*P*- value
**Width (µm)**	41.70^c^	85.53^b^	100.07^b^	131.66^a^	9.89	0.001
**Length (µm)**	197.59^c^	432.55^b^	469.00^b^	907.00^a^	77.55	0.001

Least square means (± SE) with different superscripts letters in the same row are significantly different at *p* ≤ 0.05. G1, a basal diet; G2 = a basal diet supplemented with 0.015% probiotic (Klu-zetar^®^); G3, a basal diet supplemented with 0.015% essential oil (ACTIVO^®^); G4, a basal diet supplemented with mixture of 0.015% Klu-zetar^®^ and 0.015% ACTIVO^®^, respectively

The intestinal villi showed the lowest length and width in fish fed on basal diet when compared to those fed probiotics (Klu-zetar), essential oils (ACTIVO) and mixture of probiotics and essential oils. The average intestinal villi width was (41.70 ± 9.89 μm, 85.53 ± 9.89 μm, 100.07 ± 9.89 μm, and 131.66 ± 9.89 μm) for G1, G2, G3, and G4 respectively. The average intestinal villi length was (197.59 ± 77.55 μm, 432.55 ± 77.55 μm, 469.00 ± 77.55 μm, and 907.00 ± 77.55 μm) for G1, G2, G3, and G4 respectively.

There were significant (*p* < 0.05) increases in size and thickness of the intestinal villi of *O. niloticus* fed on mixture of probiotic and essential oil than other groups (Figs. 2, 3, 4 and 5).

The scanning electron microscopy of *O. niloticus* mid intestine revealed that the intestinal villi from fish fed basal diet (G1) were the smallest of all dietary treatments; the average width was 39.1 μm as compared to those from G2, G3 and G4. The average width was 51.4 μm, 86.7 μm and 129.1 μm for G2, G3, and G4 respectively.

### Mortality rate and the relative percentage of survival (RPS) of *O. niloticus* after 7 days of challenging by *A. veronii* isolate

*O. niloticus* challenged with *A. veronii* characterized by detached scales, abdominal distension, darkness of skin, hemorrhagic patches all over the body and congestion of all internal organs (especially the kidney, liver, and spleen). The highest mortality and lowest survival rates were recorded in G1in which fish were infected with *A. veronii* and which fed on basal diet comparing to other treatment groups fed on probiotic and/or essential oil (*P* = 0.01) (Table 6).

**Table 6:** Effect of probiotic and essential oil on mortality rate and the relative percentage of survival (RPS) of Nile tilapia after 7 days post challenging by *Aeromonas veronii* isolate

Item	G1	G2	G3	G4
The number of fish/group	30	30	30	30
Mortality 1st day	10	0	4	4
Mortality 2nd day	6	2	1	3
Mortality 3rd day	4	2	2	2
Mortality 4th day	1	1	1	1
Mortality 5th day	1	0	0	0
Mortality 6th day	0	0	0	1
Mortality 7th day	0	0	0	0
Total mortality	22	5	8	11
Mortality %	73.33±5.77^a^	16.67±5.77^c^	26.67±5.77^bc^	36.67±5.77^b^
RPS %	26.67±5.79^c^	77.27±5.79^a^	63.63±5.79^ab^	50±5.79^b^

Least square means (± SE) with different superscripts letters in the same row are significantly different at *p* ≤ 0.05. G1, a basal diet; G2 = a basal diet supplemented with 0.015% probiotic (Klu-zetar^®^); G3, a basal diet supplemented with 0.015% essential oil (ACTIVO^®^); G4, a basal diet supplemented with mixture of 0.015% Klu-zetar^®^ and 0.015% ACTIVO^®^, respectively. RPS, Relative percentage of survival

## Discussion

Our current study demonstrated that incorporating dietary probiotics and essential oils into fish feed greatly improves various performance indicators, including final body weight (FBW), weight gain (WG), specific growth rate (SGR), feed conversion ratio (FCR), body length, and condition factor (CF). The highest values for these measures were observed at G2. This could be explained by the fact that probiotics and essential oils improve nutritional absorption and digestion as well as the secretion of digestive enzymes.

The current results agree with [43] who reported that the growth performance of Nile tilapia was enhanced when they were fed a basal diet supplemented with probiotics, specifically *Lactobacillus plantarum* [44]. Previous findings reported the increase of growth rates and feed efficiency in fish fed probiotics [45, 46]. Our results in line with [47] who revealed that the addition of dietary probiotics (specifically *Lactobacillus plantarum*) significantly improved the growth performance of Nile tilapia, as indicated by the final body weight, weight gain, weight gain percentage, and specific growth rate (SGR). Nile tilapia that was given diets containing varying amounts of probiotics exhibited superior growth performance indicators (such as final body weight, weight gain, average daily weight gain, and specific growth rate) compared to those fed a control diet. Probiotics efficiently inhibit the colonization of potential pathogens in the digestive system through antibiosis, competition for nutrients and space, and alterations to microbial metabolism furthermore, it improves the nutritional value by employing hydrolytic enzymes such as amylases and proteases to break down potentially indigestible food components and eliminate potentially harmful compounds present in feeds [18, 19, 48]. Common carp *(Cyprinus carpio)* fingerlings fed on diet supplemented with probiotics (*Lactobacillus fermentum*) displayed higher final body weight, weight gain, and specific growth rate and had better FCR than those fed on control basal diet [11]. Pangasius *(Pangasianodon hypophthalmus)* probiotic treated group had a higher condition factor than control group [49], this evidence is in accordance with the current study results. Contrary to our results, probiotic supplemented diets *(Bacillus sp.*,* Pediococcus sp.*,* Enterococcus sp.*,* and Lactobacillus sp.)* had no significant effect on FCR and CF of Nile tilapia [3]. The growth performance of Nile tilapia was enhanced by the addition of essential oils, as confirmed by the results of the current study [50]. In females Nile tilapia, ration supplemented with commercial essential oil compounds enhanced hepatosomatic index without any adverse effect on growth performance [51].Catfish fed on diet supplemented with essential oil exhibited a higher growth performance than fish fed on control diet [52]. The weight gain and apparent feed conversion ratio of Nile tilapia *(Oreochromis niloticus)* fingerlings were enhanced by the addition of essential oil to their diets [53]. On the other hand, Nile tilapia juveniles growth performance was not affected by addition of essential oil to their diet [9].Also, condition factor (k) of largemouth bass *(Micropterus salmoides)* was not significantly affected by essential oil supplementation [54]. The basal diet supplemented with 0,005, 0,010, 0,015 and 0,020% essential oils had no significant effect of growth of Nile tilapia as mentioned by [55].

Dietary supplementation of Nile tilapia diets with probiotics and/or essential oils had no significant effect on aspartate aminotransferase (AST), alanine aminotransferase (ALT), total cholesterol (TC), triglycerides (TG), or low-density lipoprotein (LDL). These blood biochemical indices may be influenced by other factors than diet, such as sex, general health conditions, and managemental factors. The current study revealed that fish fed a diet supplemented with essential oils had the highest high-density lipoprotein (HDL) concentrations when compared to other dietary treatments, which could be because essential oils induce hypolipidemia and hypocholesterolemia. The lower cortisol concentrations in supplemented diet groups compared to the basal control diet group in the current study served as evidence that fish diets containing probiotics and/or essential oils reduce stress. Serum biochemical parameters are widely used for estimating the health condition of fish [47], found that fish fed diets supplemented with probiotics had better health than fish fed on basal control diets. These findings are in accordance with the current study result. The current results in agreement with the findings of [11] which revealed that biochemical indices (AST, ALT, TC, and TG) of Common carp were not affected by probiotics dietary supplementation. Nile tilapia juveniles fed diets supplemented with essential oils showed higher HDL concentrations than those fed on control diets [9]. Unlike the current study, previous studies of [9, 50] which revealed that dietary supplementation of Nile tilapia fingerlings with different essential oils concentrations significantly affected AST, ALT, and TC values. Our results revealed that using diets supplemented with probiotics and / or essential oils improves stress resistance. When probiotics were administered to Nile tilapia under stressful conditions, the fish showed reduced plasma cortisol concentrations [56]. Cortisol concentrations of control group Nile tilapia were higher than essential oil treated group as mentioned by [57]. One of criteria of welfare is that the individual must free from stress in the current study the cortisol concentration were low in treated groups than the control that means using of probiotics and /or essential oils has a role in stress alleviation and keeping the internal environment of fish constant that reflect in their immunity and infection resistance hence, the mortality rates were lower in treated groups than the control one.

Regarding oxidative stress and antioxidant activity, the current results revealed that dietary supplementation of Nile tilapia with probiotics and/or essential oils enhanced the antioxidant activity and reduced oxidative stress, which was represented by low MDA, GSSG, and NO concentrations among all dietary treatment groups when compared to the control group. Furthermore, supplemented diets increase the activity of SOD, GSH, and CoQ10 that suppress oxidative stress. Probiotics and/or essential oils maintain gut health, improve metabolism, and enhance ATP and AMP concentrations. Our observation agrees with [47] who noted the reduction of MDA and NO concentrations in fish fed probiotics when compared to fish fed a control basal diet. The activity of antioxidant enzymes, such as SOD, was increased in common carp and yellow croaker fed diets supplemented with probiotics than in those fed on control diets [11, 58]. In accordance with our study, juvenile Nile tilapia supplemented with essential oils has better antioxidant activity than those without supplementation, which is expressed through low MDA levels [59, 60]. Probiotic therapy improved ATP production [61]. Contrary to the current findings, dietary probiotic supplementation had no significant effect on MDA concentrations of Nile tilapia [62]. Also, an essential oil-supplemented diet did not significantly affect SOD activity in silver catfish [7].

Intestinal histomorphology was significantly affected by dietary supplementation with probiotics and/or essential oils. The intestinal villi of Nile tilapia were increased because of dietary supplementation when compared to control diets. Probiotics and essential oils improve gut health and growth performance, which may lead to an increase in the size of intestinal villi. Intestinal morphology of Nile tilapia and rainbow trout was significantly affected by probiotics supplementation [63, 64]. After 8 weeks of probiotic feeding intestinal villi height of Nile tilapia was increased when compared to fish fed basal control diet [3]. Contrary to our result, probiotic treatment had no significant effect on intestinal villi length in Nile tilapia [65]. Feeding Nile tilapia with oregano essential oils increased the length and height of intestinal villi [53, 66].

Nile tilapia fed supplemented diets with probiotics and/or essential oils showed a higher resistance to *A. veronii* infection than those fed a basal control diet. 7 days post-infection, the total mortality number and mortality percent were high in the control group (G1) when compared to other dietary treatment groups (G2, G3, and G4). RPS % was higher in feed supplemented groups (G2, G3, and G4) than control group G1. Diets supplemented with a mixture of probiotics and essential oils are highly recommended to be used in aquaculture as they improve resistance against bacterial infection. This may be due to the protection effect of probiotics and/or essential oils against pathogenic infection. Fish immunity is increased, oxidative stress is reduced, and the gut microbiota is maintained by probiotics and/or essential oils. These factors increase fish resistance to infection. Parts of the innate and adaptive immune systems are necessary for the host to defend itself against infectious pathogens [67]. Previous studies indicated that probiotic treatments stimulate immune cell production, including leukocytes, lymphocytes, monocytes, goblet cells, and erythrocytes, and interact with immune cells to improve and incite innate immune [68, 69]. Dietary probiotic Lactobacillus plantarum improved Nile tilapia resistance to *Aeromonas sobria* infection [47]. In line with our findings, different diets supplemented with different concentrations of essential oils improved the survival rates of fish after infection with Aeromonas species [70].

## Conclusion

It is concluded that, dietary supplementations of Nile tilapia diets with probiotics and/or essential oils at a dose of 0.015% improve growth performance, enhance oxidative capacity and immunity, maintain intestinal integrity, and increase the resistance to *Aeromonas veronii* infection. In the aquaculture sector, probiotics and /or essential oils supplemented diets are highly recommended.

## Data Availability

The data presented in this study are available within the article.
